# Cleveland Clinic's summer research program in reproductive medicine: an inside look at the class of 2014

**DOI:** 10.3402/meo.v20.29517

**Published:** 2015-11-11

**Authors:** Damayanthi Durairajanayagam, Anthony H Kashou, Sindhuja Tatagari, Joseph Vitale, Caroline Cirenza, Ashok Agarwal

**Affiliations:** 1 American Center for Reproductive Medicine, Glickman Urological & Kidney Institute, Cleveland Clinic, Cleveland, OH, USA; 2 Faculty of Medicine, Universiti Teknologi MARA, Selangor, Malaysia; 3 SUNY Upstate Medical University, Syracuse, NY, USA; 4 Case Western Reserve University, Cleveland, OH, USA; 5 Northeast Ohio Medical University, Rootstown, OH, USA; 6 Faculty of Medicine, University of São Paulo, São Paulo, Brazil

**Keywords:** research training, translational medical research, biomedical research, summer internship, medical education, medical students, program development, program evaluation

## Abstract

**Background:**

The American Center for Reproductive Medicine's summer internship course in reproductive medicine and research at Cleveland Clinic is a rigorous, results-oriented annual program that began in 2008 to train both local and international students in the fundamentals of scientific research and writing. The foremost goal of the program is to encourage premedical and medical students to aspire toward a career as a physician–scientist. The internship provides participants with an opportunity to engage in original bench research and scientific writing while developing theoretical knowledge and soft skills. This study describes selected survey responses from interns who participated in the 2014 internship program. The objective of these surveys was to elicit the interns' perspective on the internship program, its strengths and weaknesses, and to obtain insight into potential areas for improvement.

**Methods:**

Questionnaires were structured around the five fundamental aspects of the program: 1) theoretical knowledge, 2) bench research, 3) scientific writing, 4) mentorship, and 5) soft skills. In addition, an exit survey gathered information on factors that attracted the interns to the program, communication with mentors, and overall impression of the research program.

**Results:**

The opportunity to experience hands-on bench research and scientific writing, personalized mentorship, and the reputation of the institution were appreciated and ranked highly among the interns. Nearly 90% of the interns responded that the program was beneficial and well worth the time and effort invested by both interns and faculty.

**Conclusion:**

The outcomes portrayed in this study will be useful in the implementation of new programs or refinement of existing medical research training programs.

The American Center for Reproductive Medicine's (ACRM) summer internship program at Cleveland Clinic (Cleveland, OH) is a 7-week annual course in recent advances in reproductive medicine. The goal of the program is to offer students a closely mentored, initial foray into the dynamic world of research in medicine. This is achieved through the objectives of the internship program, which is, for participating interns to 1) discover new knowledge from topics in reproductive medicine and other selected areas of medicine, research, and soft skills, by speakers chosen for their expertise in clinical, surgical, research, and scientific fields; 2) apply skills gathered through the daily mentoring of research work by experienced physicians and/or scientists; 3) to discover the art and science of how bench research is conducted; 4) apply skills of data compilation and statistical methods of analysis in basic science research; 5) compose the writing of scientific articles and reviews in reproductive biology and human infertility; and 6) synthesize research findings into a formal oral/PowerPoint presentation, then discuss and defend these research findings to a team of experienced scientists.

A key strength of the program is that during this intensive internship, scientists and clinicians take on roles as preceptors to mentor and teach interns the fundamental principles of bench research and scientific writing. As a whole, the internship course is founded upon five integral pillars – theoretical knowledge, hands-on bench research, scientific writing, mentorship, and soft skills. Through the internship activities, the interns obtain a foundation for a clinical or research career in medicine or its related sciences. The significance of this internship course is evident by the fact that within the past 5 years, Case Western Reserve University School of Medicine has recognized and awarded the program three times, for making a positive impact on medical education and careers of students.

With each passing year, the applicant pool has become more competitive and selective. Students accepted into the program are academically driven and were found to demonstrate a high level of intellectual curiosity based on their performance during the course of the program. Interested candidates complete an application that requires them to submit a copy of their resume and to answer a few essay-type questions that explore a variety of areas, including their interest in reproductive medicine, prior research experience, and future career plans. Most students who apply have had some form of medical or science-related educational training and/or are considering a career in medicine or research. Prior research experience is not a requirement, and in fact, most of the past interns had no previous research training.

Since the inaugural class of 2008, the program has enrolled a wide variety of students with varying academic backgrounds from all over the world. Most have been either undergraduate or medical students along with some who are completing a residency or postgraduate program. The initial course (2008) consisted of 14 students and, by the seventh annual program (2014), grew to 25. The 2014 group consisted of 3 students from the United States (12%) and 22 international students (88%).

The key features of an effective training program include the ability to provide proficient mentorship, hands-on application, teaching, and learning strategies that are tailored toward the learner and construct validity (1). However, although few articles report on and evaluate the effectiveness of research training programs, even fewer articles are available that report on the outcomes of programs that develop physician–scientists (2, 3). Thus, to gauge the effectiveness and performance of the internship course and to garner feedback from the interns in tandem with the progress of the internship, we administered surveys to assess various aspects of the internship. We hypothesized that the objectives of the internship program were achieved thorough the successful implementation of the activities centered on the five pillars of the program. These surveys were conducted periodically during the course in order to acquire real-time feedback from interns about the program's strengths and weaknesses. This survey methodology also provided us with a means to analyze and tweak the program as the internship progressed.

During the summer of 2014, a total of 12 surveys were conducted – one before the start of the internship, 10 during its 7-week duration, and one exit survey at the conclusion of the program. These survey questions were designed using questionnaires that have been administered in a comparable setting serving as a reference point. The variables chosen were those that were of our interest that would elicit student's responses about the internship in order to help improve the program. The verbiage and construction of questions have been refined through the years to be more focused on key outcome areas of the internship program. Prior to administration, all survey questionnaires were subjected to review by language and content experts who are familiar with the program.

Each survey contained between 9 and 18 questions, except for the first, penultimate, and final surveys, which had 51, 27, and 56 questions, respectively. Surveys were conducted via Google Forms (except the exit survey which was printed) and composed of qualitative questions in the form of ranking (five-point Likert scale), multiple choice, and open-ended questions.

The surveys analyzed various facets of each pillar of the program, including its logistics, implementation, and general reflections. More specifically, interns were surveyed on the pre-internship teleconferences, orientation, research communication/presentation exercises, mentors, lectures, scientific writing, bench research, and soft skills. In addition to the surveys held as the internship progressed, the comprehensive exit survey given to each intern at the end of the program was perhaps the most important of all the surveys because of the detailed information it sought on virtually every aspect of the program. Topics surveyed in the exit survey included factors that attracted the intern to the program, past experiences with scientific writing and bench research, incorporation of lecture information into scientific writing, communication with mentors, overall impression, and recommendation of the program to future candidates.

We hypothesize that students who ‘graduate’ from this internship program upon fulfilling its objectives have improved research skills because of their participation in the course. In this report, we present the outcomes of the surveys assessing various aspects of the summer internship program completed by the interns in the 2014 batch as they progressed through the 7 weeks of the internship. In addition, the goal of collecting the interns’ perspectives via these surveys was to understand the interns’ view about the program as well as to glean insight into improving the following year's program.

## Interns’ survey responses

The internship surveys were considered mandatory and, thus, required a response by all the interns within a stipulated time frame. Among the respondents, 88% (22/25) were international students who had no prior internship-type experience in the United States. All 25 (100%) interns completed each survey. Selected outcomes of the surveys, which are categorized based on the five core pillars of the program, are presented here. Following this, survey responses regarding the pre-internship teleconference calls and feedback in the exit survey that summarizes the internship program are highlighted.

### Lecture-based theoretical knowledge

Over the first 5 weeks of the internship, lectures were delivered by Cleveland Clinic faculty as well as invited scientists and/or clinicians. The lecture component covered a wide range of topics, from those related to reproductive medicine (with a focus on infertility) through the basics of conducting bench research and scientific writing, to public speaking and experience sharing (Table 1). The aim of these lectures were to lay the foundation for the interns’ theoretical understanding of forthcoming research projects and thus facilitate their participation in scientific writing and bench research. These lecture-based sessions were mostly interactive, consisting of an open forum discussion throughout the presentations followed by a 10-min question and answer session.

**Table 1:** Summary of 2014 summer internship program lecture topics and teaching hours

Topics		Lectures	Workshop/mini-symposium	Hands-on training/mentoring and research work	Total hours
1.	Male fertility	10	–	–	10
2.	Female fertility	6	6	–	12
3.	Human fertility and others	2	–	–	2
4.	Scientific writing and training	9	4	82	95
5	Bench research and training	4	–	116	120
6	Public speaking and training	1	–	29	30
7	Personal and professional development	7	–	–	7
8	Cleveland Clinic and American Center for Reproductive Medicine	4	–	–	4
	Total hours	43	10	227	280

In general, responses to the surveys demonstrated that the lectures were beneficial in familiarizing the interns with the multiple layers of the program. Of equal importance to the interns were the talks given by scientists and clinicians on various reproductive physiology and pathology topics. Nearly all interns found these lectures valuable in furthering their understanding of their research assignments and applying their scientific knowledge to a bench research project.

**Fig. 1:**
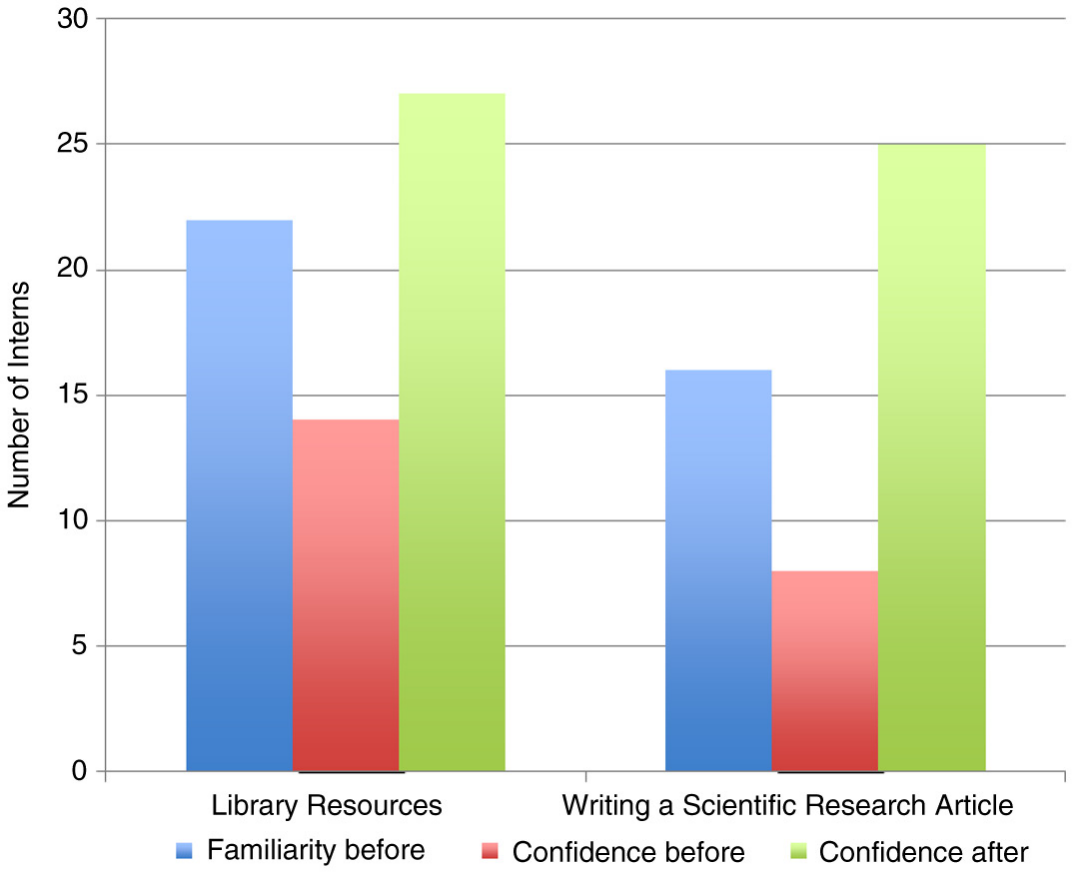
Familiarity and confidence levels of interns regarding performing a library search and writing a scientific research article before and after the scientific writing lecture series during the 2014 summer internship course.

Among the lectures delivered on scientific writing, ‘library resources’ and ‘how to write an informative and concise, yet interesting title, abstract, and introduction’ were found to be most useful by 64% (16/25) of the interns. When asked about the usefulness of the talk that discussed how to conduct an exhaustive literature search on a scientific writing topic, most of the interns responded that they had previously heard of the process and search engines, but lacked confidence in completing the database search on their own. However, after several training sessions from the faculty and librarians, the interns felt that the step-by-step exercise gave them more confidence and clarity in conducting the database search without assistance (Fig. 1). Similarly, after several workshop-like sessions with the manager of medical editing services, the interns became more accustomed with the writing process and were comfortable working independently.

Furthermore, the lectures on presentation skills, leadership and team work, and time-management skills helped boost the interns’ confidence when presenting their respective research projects, working together as a team in a culturally diverse group, and managing the intense internship workload, respectively. These soft skills were nurtured and refined throughout the internship through its daily activities (*see Soft Skills section*).

Besides theoretical knowledge, the lecture series also exposed the interns to prominent clinicians and scientists who continue to contribute passionately to the medical profession. In their response, interns acknowledged that the additional advantage of these talks was that it created the opportunity for them to interact directly with expert clinicians and investigators in a classroom setting. This exposure also served as further motivation in the interns' early career, as well as demonstrated the significance of physician–scientist in the advancement of medicine. One intern stated, ‘Hearing the lectures from the various esteemed faculty was very inspiring as we got a peek into their vision and innovation’.

### Hands-on original bench research

**Fig. 2:**
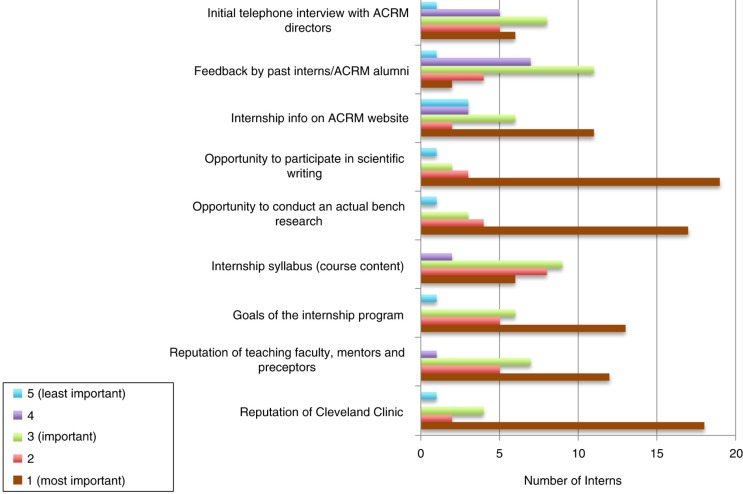
Factors that influenced the 2014 interns’ interest in joining the summer internship program.

Another important pillar of the program is the hands-on bench research. More than 68% (17/25) of interns chose the opportunity to participate in an original bench research project as one of the most important factors when deciding to apply to the program (Fig. 2). Based on their past laboratory experience, interns were allocated into teams to work on different original bench research projects. These original projects had been specifically designed by the organizing faculty/mentors for the purpose of a team research effort during the internship.

Under the direct supervision of a reproductive biologist/researcher who serves as the lead mentor for their assigned project, interns undergo vigorous training to become proficient at performing the relevant techniques pertaining to their project. Before they can qualify to assist in the actual research project and conduct the experiments, interns must have achieved a sound understanding of the project's basis and goals, and verification by their project mentor and the program director. This research opportunity encourages interns to use their background knowledge to solve current clinical problems. Furthermore, by performing bench work, interns are driven to progressively develop leadership, teamwork, and time management skills (*see Soft Skills section*).

**Fig. 3:**
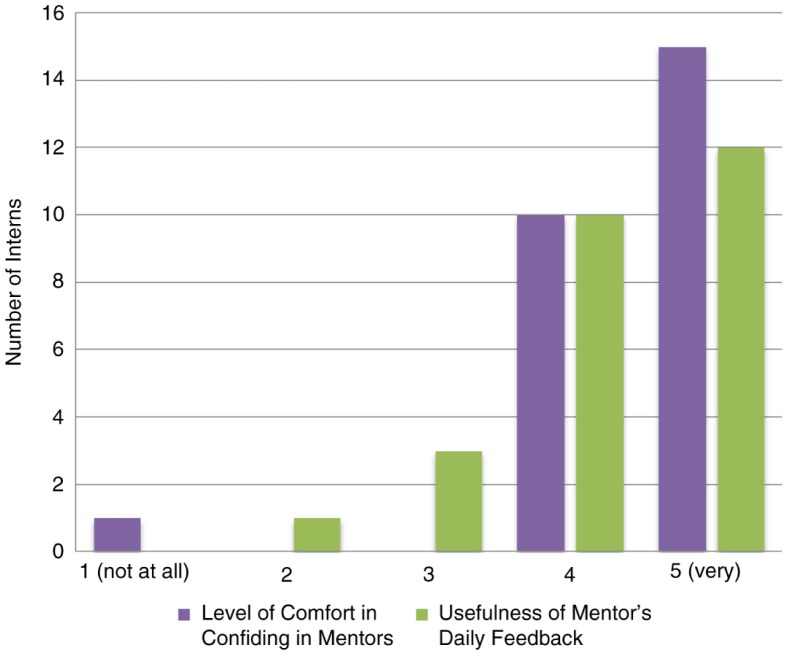
Level of comfort in confiding in mentors and usefulness of mentor's daily feedback.

A detailed questionnaire surveyed the interns on their bench research experience. Most noted the mentorship they received and the ability to work as a team as the two most valued qualities of the experience. Mentors trained interns in all the required research techniques, assisted throughout all aspects of the research project, and guided them in preparing for their presentations. Nearly all interns (24/25, 96%) were either more than or very comfortable in confiding in their mentors regarding the difficulties that they faced while conducting bench work (Fig. 3). All interns found the daily feedback from mentors to be very useful (12/25, 48%), more than useful (10/25, 40%), and useful (3/25, 12%) (Fig. 3). Almost all interns mentioned that the mentors’ assistance (24/25, 96%) and the presentations (22/25, 88%) helped them achieve a greater understanding of their research topics (*see Mentorship section*).

Furthermore, interns realized the importance of working as a team in all aspects of the project. Without the proper communication and willingness to work together, the interns believed that their bench research results would be compromised and that the day would drag on late into after hours. Teamwork was identified as the most vital aspect of the project's success. Time management also became an essential factor, particularly on bench research days. Interns found that they not only had to be prepared at all times but also manage their other tasks, such as presentations, while maintaining smooth progress with their writing assignment. Overall, interns rated the hands-on bench research experience as one of the most rewarding aspects of the program.

### Scientific writing

One of the most important aspects of the scientific process is the ability to communicate knowledge in a concise and effective manner. Another major pillar of the summer internship program is scientific writing for publication. In fact, 76% (19/25) of interns chose this as one of the most important factors when deciding to apply to the program (Fig. 2). Interns are presented with the opportunity to share their knowledge with the scientific community through writing a research article, mainly in the form of a review article or a book chapter. By participating in this writing exercise, interns not only learned the scientific writing process but also gained a potential opportunity to publish their work.

**Fig. 4:**
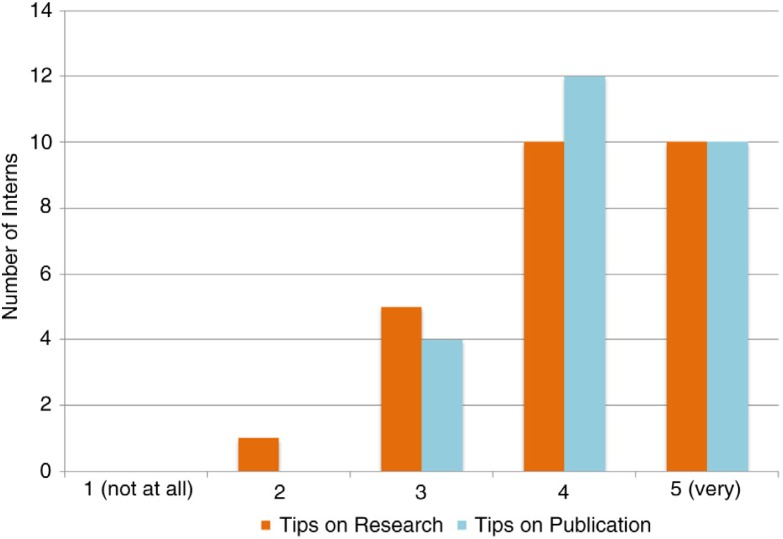
Usefulness of lectures sharing tips on research and publication.

A series of lectures given in the first few weeks provided the foundation of how to research, write, and present their topic. Besides theoretical lectures on scientific writing, the interns also participated in sharing sessions with the mentors, who shared their own perspectives, experiences, and tips through talks focusing on research and publications. Most interns found these topics useful or very useful (an average of 21/25, 84%) (Fig. 4). All interns are assigned a writing topic in reproductive medicine as well as a lead mentor who personally assists, supports, and guides them throughout the scientific writing process. Frequent updates and one-on-one meetings with the mentors are required to assess progress, provide real-time feedback and advice, and strategize how to move forward. This self-directed learning project gives interns a chance to analyze a complex topic, think critically, and uniquely contribute their findings toward existing scientific literature. Mentors scaffold the interns’ learning and motivate them to carry out their tasks until the project goal is achieved (*see Mentorship section*).

Interns felt that the related lectures, one-on-one mentoring, and opportunities to deliver oral presentations on their topics assisted them in their understanding of the subject and helped them successfully complete this important undertaking for the first time. From their first 2-min presentation, interns responded that the factors they had found most difficult to do were 1) summarizing the large amount of information on their topic within the time limit given (44%, 11/25); 2) time management (40%, 10/25); and 3) not fidgeting/not standing frozen in front of an audience (24%, 6/25). Only 8% (2/25) interns responded that it was most difficult for them to hear/receive feedback from all the mentors/fellow interns. One of the interns stated, ‘I am excited about the next presentation because I hope to do much better than this time’. By the end of the internship, nearly all interns responded that the ability to contribute to the medical field in some way through their writing was a great feeling and accomplishment.

### Mentorship

One of the most straightforward approaches to encourage and promote translational research is to promote the structured mentoring and training of prospective researchers (4). For this reason, the program's one-on-one mentoring by the internship faculty remains one of the most sought after and unique features of the program. Our mentors come from different parts of the world and bring with them a wealth of experience from clinical, academic, and research backgrounds. These mentors have received extensive training in their respective fields and are well-acquainted with assisting graduate- or postgraduate-level students. In fact, 48% (12/25) of interns chose the reputation of the teaching faculty/mentors as one of the most important factors when deciding to apply to the program (Fig. 2).

Scientific writing and bench research are two enormous tasks that are undertaken by the interns. The mentors guide and evaluate the interns throughout their scientific writing and bench research projects and for their oral presentations of their projects. These exercises provide interns with real-time constructive feedback, which helps strengthen their understanding of their research topic and stay on track with their scientific communication and in their work moving forward. Furthermore, the oral presentation exercises and subsequent personalized feedback by mentors allow the interns to improve their ability to effectively communicate their scientific findings and speak in front of an audience (*see Soft Skills section*).

This integrated and individualized mentorship approach, in which doctoral-level educators enthusiastically invest time and energy in each intern, provides much of the integral basis for the program's continued success. Students from Generation Y (born between early 1980s to early 1990s), as these interns are, prefer working under the supervision of those who are approachable and supportive as well as good communicators and motivators (5). A good mentor possesses the ability to shape an intern's experience. The mentors involved in this program place a lot of personal effort into the training and hands-on conduct of bench work, performing extensive literature searches and reviews, and writing and revising manuscript drafts with their respective interns. From the interns’ perspective, the guidance and mentorship played an important role in the success of each intern's scientific writing and bench research projects (Table 2).

**Table 2:** Interns’ descriptions of the kind of mentoring they received during their bench research training

Intern	Response regarding mentor/mentoring received
1	‘She was teaching us each test clearly and understandably, motivating with her knowledge’
2	‘She was very helpful with everything, gave us proper training and answered all of our questions’
3	‘He is a very good teacher and easily approachable, he had no issues with discussing everything more than once if we required, he was always checking to make sure we knew what we were doing and made sure we were doing it right’
4	‘He helped me organize my thoughts by listening to me and correcting whatever ideas or point I get wrong, he encourages me to work and to submit work on time’
5	‘He described our objectives first, then he explains to us how to do it, after that he gave us the opportunity to try it ourselves with his help’
6	‘My Mentor really paid attention to involve everyone in everything and helped us to improve in every technique’
7	‘He one of the best mentors and persons I have ever met, he is very enthusiastic about his or I should say ‘our’ project, he is always there to help us out, he is really friendly and is always willing to teach us extra stuff’
8	‘Supportive, helpful, and just a comfortable presence’
9	‘I was not afraid to ask questions and the mentoring was perfect’
10	‘Excellent! He always explains everything and re-explains if I don't understand something. A wonderful mentor’

Prior to presentations, the mentors played a key role in assisting interns in their preparations. Most students relied on or consulted with their mentors for assistance and suggestions. Before their bench research presentation, interns responded that presenting among their team members (16/25, 64%) and then to the mentor as a team (12/25, 48%) and receiving feedback was beneficial. The preparation with their mentors made them more comfortable in presenting and they felt that they did well for their actual presentation after practicing once with their mentor. One intern responded, ‘My mentor was really helpful in every stage. He listened to our presentation, gave feedback, helped us find resources, and was very encouraging, giving me confidence for the presentation’. Most of the interns (24/25, 96%) felt that the mentors were very helpful in guiding them for their presentations. When asked what their mentors could do to help further improve the interns’ performance, the interns’ response was ‘nothing’ and ‘just continue doing what they are doing’.

### Soft skills: communication, public speaking and others

Soft skills are a collective term for skills such as leadership, communication, interpersonal skills, attitude, and work ethics, which help an individual work and socialize in a positive manner. Soft skills such as these complement the individual's hard skills, which are his/her knowledge and occupational/technical skills. Effective soft skills are much sought after in the workforce for their contributory role in career growth and as a distinguishing factor of an individual's caliber among his/her peers. In research and medicine, prominence is placed on effective communication and interpersonal skills. Presentation and public speaking skills are extremely valuable commodities, particularly in medical research, as they represent an ability to disseminate scientific knowledge in a clear, concise, and methodical manner. Moreover, the concepts of professionalism and teamwork are given equal emphasis, in order to guarantee outcomes of the highest quality.

The internship offers several lectures to the interns on these topics, providing them with valuable information and opportunities to build on each of these skills. The interns surveyed thought that the information on public speaking was most helpful (11, 44%). Although these concepts were not entirely new to the interns in this stage of their studies (Table 3), there were suggestions and assets that they acquired from these lectures. Several students emphasized the importance of ‘preparation, organization, and interaction with the audience’ in public speaking, all of which are key features of a successful and memorable presentation.

**Table 3:** Interns’ application and perception of new information gained from selected soft skills lecture topics

	Application during internship			Interns’ perception: percentage of new information they learnt through the lectures				
			
Lecture topics	Scientific writing	Bench research	Application beyond internship	100% (everything was new)	75%	50%	25%	0% (nothing new)
Public speaking	✓	✓	✓	8% (n=2)	16% (n=4)	24% (n=6)	36% (n=9)	16% (n=4)
Dialogs in professionalism	✓	✓	✓	8% (n=2)	32% (n=8)	24% (n=6)	32% (n=8)	4% (n=1)
Teamwork and team building	✓	✓	✓	4% (n=1)	36% (n=9)	12% (n=3)	32% (n=8)	16% (n=4)

As was described previously, several oral and PowerPoint presentations for both scientific writing and bench research were assigned periodically during the internship. These presentations provided a summarized look at each project and helped solidify the interns’ comprehension of their research topics. During these sessions, interns presented their research topic and the progress made, while all the mentors evaluated them and individually provided suggestions for improvement. Many, if not all, interns found the feedback given to them on their presentation and delivery style to be beneficial. One student noted, ‘The feedback was spot on, as they were critical and correct. They praised all the good qualities in me but at the same time criticized qualities which I needed to improve on’. The suggestions obtained from each of the mentors were applied in the interns’ next oral presentation, thus demonstrating the continuous impact of the mentorship process. In addition to mentor feedback, fellow interns were also encouraged to share their thoughts on their peers’ performance, which was found to be helpful.

Most of the interns stated that they became more comfortable with public speaking because of these presentations. Furthermore, 96% (24/25) found this to be a valuable experience. As such, all (100%) thought that having additional time to practice would have improved their performance. Hence, adjustments to allow more time for practice sessions were taken into account in the following year's internship schedule (in 2015).

Personalized mentorship remains a striking feature of the internship experience. Interns and mentors must foster clear channels of communication, both in written and verbal form, in order to forge successful, lasting relationships. Efficient communication between the interns and mentors enhanced the interns’ success and experience, whether in the laboratory, during writing, or in other activities of the program. As stated by an intern, ‘Our environment (in the laboratory) is low pressure and efficient because the mentor and the interns have a good relationship’. Exchange of frequent and consistent updates (interns to mentors) and feedback (mentors to interns) was essential to ensuring that the interns successfully completed their bench and writing projects. Individual meetings with mentors on scientific writing were found to be extremely helpful as well. During these meetings, the interns updated their mentor on any findings, revisions, and plans on moving forward.

Bench research makes up a significant portion of the internship curriculum. Teamwork was vital in completing the projects in an efficient and orderly manner, particularly in the bench research activities and presentations, which entailed unified work in a small group. Many interns reiterated that working as a cohesive unit and the constant involvement of their peers substantially improved teamwork as a whole.

### Internship teleconference

Two months prior to the start of the internship, two teleconferences were held – 1 month apart – involving all the accepted interns from their respective home cities and the faculty/mentors at Cleveland Clinic. The purpose of the first teleconference was to acclimate the interns to the structure, policies, and expectations of the program whereas the second delved into more details regarding the curriculum and day-to-day logistics. The meeting agenda was provided ahead of time to allow interns to be aware of the topics discussed and prepare any questions that they may have. Responses from the interns (22/25, 88%) indicated that this was helpful in providing a well-organized, focused, and purposeful discussion.

Throughout each teleconference, students responded that they felt encouraged to freely ask questions (24/25, 96%) and convey any apprehension on their part. The interns unanimously (25, 100%) indicated that these calls helped to alleviate any fear and uneasiness that they may have had regarding the upcoming program. As a result of the teleconferences, all 25 interns (100%) felt that they were able to better associate themselves with their future mentors and the program's general structure.

All 25 interns responded that the calls showcased the quality of their prospective mentors and helped to increase their understanding and expectations of the program. In addition, 92% (23/25) found that the teleconference calls presented a helpful avenue to connect with their fellow interns in advance of the internship.

We reiterate here that communication between mentors, interns, and the course coordinators is crucial to the success of any program. The preferred methods of communication for 80% (20/25) of the interns were e-mail (uniformly Gmail) and WhatsApp. These efficient means of communication allowed for timely instructions and updates to be communicated not only throughout the internship but also prior to the program.

Overall, the teleconference meetings allowed the interns to feel more comfortable (20/25, 80%) prior to their arrival and that they were going to be in good hands (19/25, 76%). Furthermore, their interaction with the faculty during the teleconference increased the interns’ confidence in their mentors (20/25, 80%) and emphasized the significance of the internship program (19/25, 76%) from early on.

### Internship orientation

The first day of any educational program is a crucial one, as this is when students formulate their foremost impressions of their surroundings, colleagues, and the program itself. During the program's orientation process, a structured, hour-by-hour daily schedule was distributed along with other essentials. This schedule was carefully designed months ahead of the program's start date in order to facilitate smooth progression of activities over the course of the 7 weeks. Prior to obtaining the physical copy of the internship materials, an electronic copy was sent to the interns, which allowed them early access to important internship information. One intern stated, ‘I really liked how everything is really organized and prepared for us in advance’. The entire class (25, 100%) believed it was beneficial to have the internship material beforehand, as this allowed interns to prepare their thoughts and provided them with information on what to expect from the program.

Prior to their arrival, interns had to also read and sign a policy document explaining the ACRM's policies and procedures. All interns (25, 100%) believed this to be helpful and informative as it allowed them to have a basic understanding of what was expected of them from the start. More than 95% (24/25) of the students thought that the orientation process was streamlined and well structured. One intern described the orientation experience as, ‘It was a smooth and comfortable process, and the staff was very welcoming’. Other interns were of the opinion that the orientation sessions were ‘helpful’, ‘well organized’, ‘detailed’, and ‘thorough’. This feedback helped to validate the effectiveness of the orientation process.

### Exit survey

Cleveland Clinic is a world-renowned medical institution that is consistently recognized for its commitment to excellence and leadership in both the practice of medicine and in research innovation. Interns were surveyed on aspects that contributed to their decision to apply to the program (Fig. 2). Cleveland Clinic is recognized as one of the top five hospitals in the United States, and its teaching faculty, mentors, and preceptors were consistently ranked (18/25, 72%) as major factors. More than half of the students who attended the internship did not have any previous experience in scientific writing (22/25, 88%) and hands-on bench research (17/25, 68%). However, the opportunity to participate in both scientific writing and bench research was a powerful determinant of their interest in attending the program. The goals of the internship program (13/25, 52%) and the information provided on the ACRM website (11/25, 44%) were also mentioned as significant reasons for choosing to attend the internship program.

The exit survey also looked at the overall experience of the student, asking them to describe the internship as excellent, very good, good, fair, or poor. A resounding number of interns (22/25, 88%) rated their experience in the category of excellent, very good, or good. Given this, it can be inferred that the common viewpoint of more than 80% of the class regarding the program was a positive one. And as with any other course that provides an educational service, one of the inherent factors to look at is the target audience's overall impression of the service offered. Thus, it is reassuring to know that 88% (22/25) of the interns found the program to be beneficial and well worth the time and dedication, on both the part of the interns and the faculty.

## Advantages and limitations

One of the main reasons why the surveys were conducted as compulsory questionnaires throughout the course is that, in spite of the success of any program, there is always room for improvement. Based on their interview at the time of joining the course, interns expected through their participation in the internship to gain knowledge and/or skills in: topics in reproductive and other selected areas of medicine, research, and soft skills; daily mentoring of research work by experienced scientists; the conduct of bench research and data collection; writing of scientific articles or reviews in reproductive biology and human infertility; and being able to summarize, discuss, and defend their research findings, then present these findings in a formal oral/PowerPoint presentation to a team of experienced scientists. These interns’ expectations aligned well with our course objectives.

Information garnered from the survey responses proved our hypothesis and demonstrated the program's effectiveness in mentoring and training students in scientific writing, bench research, and effective communication, thus fulfilling the interns’ expectations from participating in the program. Responses from these surveys gave a sense of overall impressions of the interns, allowed for the strengths and weaknesses of the program to be captured, and generated suggestions for improvement in upcoming programs. By scrutinizing the collective responses of the interns, the ACRM team was able to use the feedback to refine the program. For example, several interns had expressed dissatisfaction about having too many lectures and not enough time for self-study to work on scientific research and bench research. This feedback was taken into consideration in the planning of the 2015 ACRM summer internship program, and the number of lectures was significantly decreased. Timely feedback of the interns’ perspectives was valuable in helping lead the program along an enhanced and effective route.

The advantages of collecting participant feedback are that it is cost effective, is relatively straightforward to carry out, and can potentially be completed in a timely manner by all participants. The disadvantage of these types of surveys is that the respondents may feel encouraged or obliged to provide positive answers that do not reflect their true thoughts, or rather, provide answers that present themselves in a favorable manner to the faculty. It is also very easy to have bias within a survey, depending on the respondents. A major challenge was that the interns became tired of completing the numerous surveys within a short span of time, and some required reminders to complete them on time.

## Significance and going forward

A proud achievement of the program is that nearly all past medically qualified international interns that went on to apply to a residency program in the United States have received a positive outcome. These interns themselves have attributed their participation in this internship program as a major determinant of their success because of the interest it generated during their interviews for residency positions. This is partially due to the fact that, to the authors’ knowledge, there is no other program currently in the country that allows for an intern to be intensively engaged in the conduct of both bench research and scientific writing during a relatively short period of time. Being a published individual is an admirable feat, and the fact that this internship provides the opportunity for an intern to participate in mentored scientific writing from conception to publication surpasses other similar programs.

In conclusion, this study has described how participation in our internship provides the opportunity for interns to gain knowledge and/or skills in current topics in medicine and research from renowned speakers, experience effective daily research mentoring by resourceful and results-oriented mentors, engage in hands-on conduct of bench research and data collection, experience the writing of novel scientific research, and hone their presentation skills. Based on the responses highlighted in this study, we highly recommend that a similarly-structured mentorship program be implemented in other branches of medicine or medical sciences, in order to present more opportunities for students to gain significant exposure to research at an early stage of their careers.

To the authors’ knowledge, the program's goal of offering students an insight into the vast area of research in medicine has not been accomplished in the same intensity and success by any other program. The ACRM summer internship program at Cleveland Clinic continues to grow and be refined each year, and the applicant pool has become more competitive and selective, despite an increase in the number of interns accepted per class. As the program goes into its ninth year in 2016, and more recognition is being awarded to the program, it is convincing that the program has a bright future ahead of itself.
